# Prediction of an arc-tunable Weyl Fermion metallic state in Mo_*x*_W_1−*x*_Te_2_

**DOI:** 10.1038/ncomms10639

**Published:** 2016-02-15

**Authors:** Tay-Rong Chang, Su-Yang Xu, Guoqing Chang, Chi-Cheng Lee, Shin-Ming Huang, BaoKai Wang, Guang Bian, Hao Zheng, Daniel S. Sanchez, Ilya Belopolski, Nasser Alidoust, Madhab Neupane, Arun Bansil, Horng-Tay Jeng, Hsin Lin, M. Zahid Hasan

**Affiliations:** 1 Department of Physics, National Tsing Hua University, 30013 Hsinchu, Taiwan; 2 Laboratory for Topological Quantum Matter and Spectroscopy (B7), Department of Physics, Princeton University, Princeton, 08544 New Jersey, USA; 3 Centre for Advanced 2D Materials and Graphene Research Centre, National University of Singapore, 6 Science Drive 2, 117546 Singapore, Singapore; 4 Department of Physics, National University of Singapore, 2 Science Drive 3, 117542 Singapore, Singapore; 5 Department of Physics, Northeastern University, Boston, 02115 Massachusetts, USA; 6 Condensed Matter and Magnet Science Group, Los Alamos National Laboratory, Los Alamos, 87545 New Mexico, USA; 7 Institute of Physics, Academia Sinica, 11529 Taipei, Taiwan; 8 Princeton Center for Complex Materials, Princeton Institute for the Science and Technology of Materials, Princeton University, Princeton, 08544 New Jersey, USA

## Abstract

A Weyl semimetal is a new state of matter that hosts Weyl fermions as emergent quasiparticles. The Weyl fermions correspond to isolated points of bulk band degeneracy, Weyl nodes, which are connected only through the crystal's boundary by exotic Fermi arcs. The length of the Fermi arc gives a measure of the topological strength, because the only way to destroy the Weyl nodes is to annihilate them in pairs in the reciprocal space. To date, Weyl semimetals are only realized in the TaAs class. Here, we propose a tunable Weyl state in Mo_*x*_W_1−*x*_Te_2_ where Weyl nodes are formed by touching points between metallic pockets. We show that the Fermi arc length can be changed as a function of Mo concentration, thus tuning the topological strength. Our results provide an experimentally feasible route to realizing Weyl physics in the layered compound Mo_*x*_W_1−*x*_Te_2_, where non-saturating magneto-resistance and pressure-driven superconductivity have been observed.

## Abstract (web summary)

Two-dimensional materials provide possible platforms to map exotic phenomena from particle physics to condensed matter physics. Here, the authors predict doping dependent electronic behaviour in Mo_*x*_W_1−*x*_Te_2_, in resemblance of a phenomenon which is believed to exist only in particle physics.

## Graphical abstract

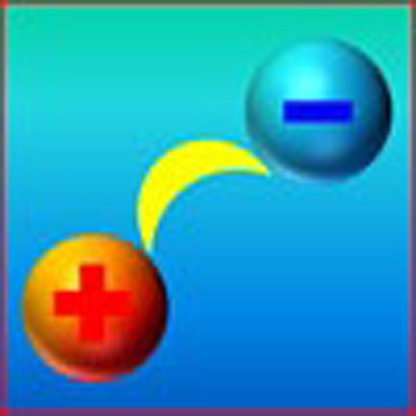

Research on Dirac fermions in graphene and topological insulators have been one of the main themes in condensed matter physics and materials science in the past decade. A particle that is also relevant for both condensed matter and high-energy physics and that may give rise to even more exotic physics is the Weyl fermion. The Weyl fermion was theoretically discovered by Weyl^1^ in 1929. He noted that the Dirac equation takes a simple form if the mass term is set to zero: 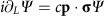, where *c* is constant, 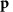 is momentum, 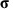 is conventional Pauli matrices and *Ψ* is wavefunction. Such a particle, the Weyl fermion, is massless but is associated with a chirality. Weyl fermions may be thought of as the basic building blocks for a Dirac fermion. For example, two Weyl fermions of opposite chirality can combine to form a massless Dirac fermion. Weyl fermions have played a vital role in quantum field theory but they have not been found as fundamental particles in vacuum. A Weyl semimetal is a solid-state crystal that host Weyl fermions as its low-energy quasiparticle excitations^1^,^2^,^3^,^4^,^5^,^6^,^7^,^8^,^9^,^10^,^11^,^12^,^13^,^14^,^15^,^16^,^17^. Weyl semimetals have attracted intense research interest not only because they provide a Weyl fermion in nature, but also because they allow topologically nontrivial states in metals not insulators. In a Weyl semimetal, a Weyl fermion is associated with an accidental degeneracy of the band structure. Away from the degeneracy point, the bands disperse linearly and the spin texture is chiral, giving rise to a quasiparticle with a two-component wavefunction, a fixed chirality and a massless, linear dispersion. Weyl fermions have distinct chiralities, either left-handed or right-handed. In a Weyl semimetal crystal, the chiralities of the Weyl nodes give rise to topological charges, which can be understood as monopoles and anti-monopoles of Berry flux in momentum space. Remarkably, the topological charges in a Weyl semimetal are protected only by the translational invariance of the crystal^6^,^8^,^17^. The band structure degeneracies in Weyl semimetals are uniquely robust against disorder^6^,^8^,^17^, in contrast to the Dirac nodes in graphene, topological insulators and Dirac semimetals, which depend on additional symmetries beyond the translational symmetry^13^,^14^,^15^,^16^,^17^,^18^. As a result, the Weyl fermion carriers are expected to transmit electrical currents effectively. Moreover, the transport properties of Weyl semimetals are predicted to show many exotic phenomena including the negative magnetoresistance due to the chiral anomaly known from quantum field theory, non-local transport and quantum oscillations where electrons move in real space between opposite sides of a sample surface^10^,^11^,^12^. These novel properties suggest that Weyl semimetals are a flourishing field of fundamental physics and future technology. The separation of the opposite topological charges in momentum space leads to surface state Fermi arcs which form an anomalous band structure consisting of open curves that connect the projections of opposite topological charges on the boundary of a bulk sample. Within band theory, the only way to destroy the topological Weyl phase is to annihilate Weyl nodes with opposite charges by bringing them together in the reciprocal space. Thus the length of the Fermi arc provides a measure of the topological strength of a Weyl state.

For many years, research on Weyl semimetals has been held back due to the lack of experimentally feasible candidate materials. Recently, a family of isostructural compounds, TaAs, NbAs, TaP and NbP, was theoretically predicted and experimentally discovered as the first Weyl semimetals^19^,^20^,^21^,^22^,^23^,^24^,^25^,^26^,^27^,^28^. So far, the TaAs class of the four iso-electronic compounds remains to be the only experimentally realized Weyl semimetals^21^,^22^,^23^,^24^,^25^,^26^,^27^,^28^.

Tungsten ditelluride, WTe_2_, is a member of the transition metal dichalcogenide materials that has recently drawn significant interest because it shows very large, non-saturating magnetoresistance and pressure-induced superconductivity^29^,^30^,^31^,^32^,^33^,^34^. It has an inversion symmetry breaking crystal structure, and exhibits a compensated semi-metallic ground state^29^,^31^,^32^,^33^. The coexistence of inversion symmetry breaking and semi-metallic transport behaviour resembles the properties of TaAs and hence suggests a possible Weyl semimetal state. Here, we propose a tunable Weyl metallic state in Mo-doped WTe_2_ via our first-principles calculation, where the length of the Fermi arc and hence the topological strength of the system can be adiabatically tuned as a function of Mo doping. A very recent paper^35^ predicted the Weyl state in pure WTe_2_, but the separation between Weyl nodes was reported to be beyond spectroscopic experimental resolution.

In this paper, we demonstrate that a 2% Mo doping is sufficient to stabilize the Weyl metal state not only at low temperatures but also at room temperatures. We show that, within a moderate doping regime, the momentum space distance between the Weyl nodes and hence the length of the Fermi arcs can be continuously tuned from 0 to ∼3% of the Brillouin zone (BZ) size via changing Mo concentration, thus increasing the topological strength of the system. Our results present a tunable topological Weyl system, which is not known to be possible in the TaAs class of Weyl semimetals.

## Results

### Material system considerations

**Figure 1:**
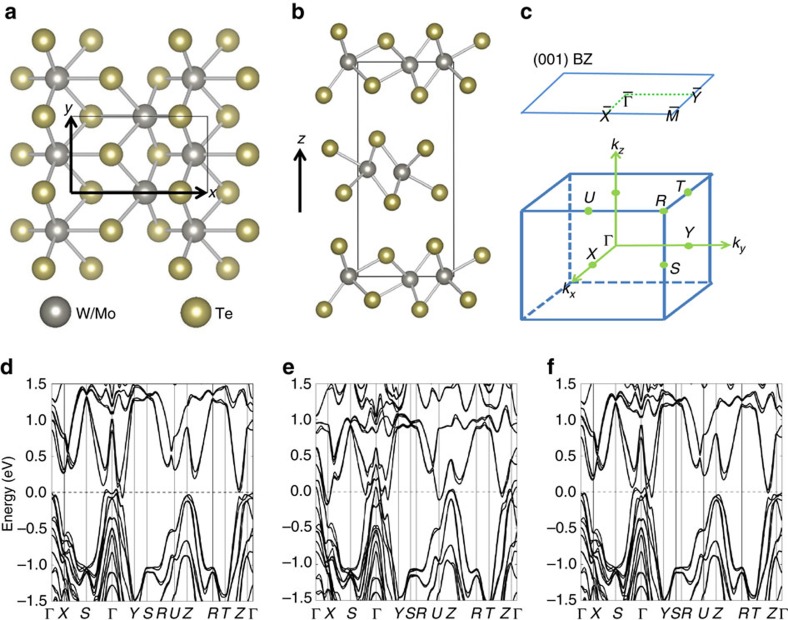
Crystal structure and band structure. (**a**) A top view of WTe_2_ lattice. (**b**) A side view of WTe_2_ lattice. The silver and yellow balls represent W and Te atoms, respectively. Rectanglar box denotes crystal unit cell. WTe_2_ crystalizes in an orthorhombic Bravais lattice, space group *Pmn*2_1_ (No. 31). The lattice constants are *a*=6.282 Å, *b*=3.496 Å, *c*=14.07 Å according to a previous X-ray diffraction measurement^36^. (**c**) The bulk and (001) surface BZ of WTe_2_. (**d**) Bulk band structure of WTe_2_. (**e**) Bulk band structure of MoTe_2_ by assuming that it has the same crystal structure as WTe2. Note that, in fact, according to available literature MoTe_2_ has two possible structures^36^,^37^, both of which are different from the crystal structure of WTe_2_. (**f**) Bulk band structure of Mo_0.2_W_0.8_Te_2_.

WTe_2_ crystalizes in an orthorhombic Bravais lattice, space group *Pmn*2_1_ (No. 31). In this structure, each tungsten layer is sandwiched by two tellurium layers and forms strong ionic bonds. Figure 1a shows a top view of the lattice. It can be seen that the tungsten atom is shifted away from the centre of the hexagon formed by the tellurium atoms. This makes the in-plane lattice constant along the 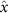 direction *a* longer than that of along the 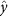 direction *b*. The WTe_2_ sandwich stacks along the out-of-plane 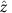 direction, with van der Waals bonding between layers (Fig. 1b). We used the experimental lattice constants reported by Brown^36^, *a*=6.282 Å, *b*=3.496 Å, *c*=14.07 Å. The bulk BZ and the (001) surface BZ are shown in Fig. 1c, where high symmetry points are noted. In Fig. 1d, we show the bulk band structure of WTe_2_ along important high symmetry directions. Our calculation shows that there is a continuous energy gap near the Fermi level, but the conduction and valence bands have a finite overlap in energy. The band gap along the Γ−*Y* direction is much smaller than that of along the Γ−*X* direction or Γ−*Z* direction, consistent with the fact that the lattice constant *b* is much smaller than *a* and *c*. At the Fermi level, our calculation reveals a hole pocket and an electron pocket along the Γ−*Y* direction (Fig. 1d), which agrees with previous calculation and photoemission results^29^,^31^,^32^,^33^. We also calculated the band structure of MoTe_2_ by assuming that it is in the same crystal structure. As shown in Fig. 1e, the general trend is that the bands are ‘pushed' closer to the Fermi level. For example, in MoTe_2_, there are bands crossing the Fermi level even along the Γ−*X* and Γ−*Z* directions. We emphasize that, according to available literature^36^,^37^, MoTe_2_ has a different crystal structure, either hexagonal^37^ or monoclinic^36^ both of which have inversion symmetry, but not orthorhombic. Thus in our calculation we assumed that MoTe_2_ has the orthorhombic crystal structure as WTe_2_ and obtained the lattice constants and atomic coordinates from first-principle calculations. Very recently, a paper^38^ claimed that MoTe_2_ can be grown in the orthorhombic structure. This still needs to be further confirmed.

**Figure 2:**
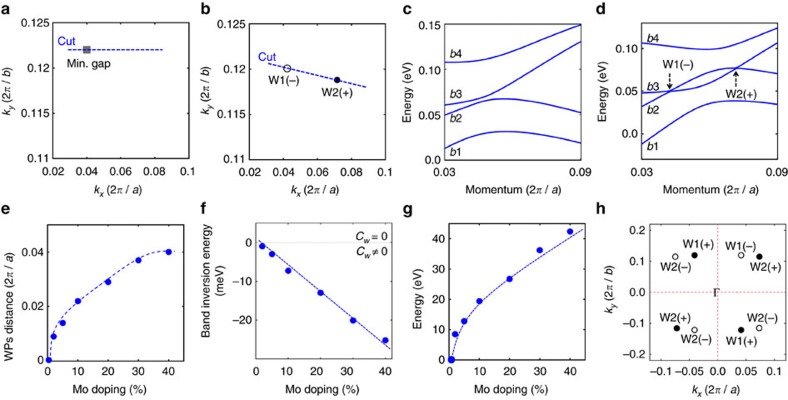
Tunable Weyl node separation (topological strength) in Mo-doped WTe_2_. (**a**) The *k* space location that corresponds to the minimal energy gap of the pure (undoped) WTe_2_. (**b**) The *k* space locations of the pair of Weyl nodes within the first quadrant of the *k*_*z*_=0 plane for Mo_0.2_W_0.8_Te_2_. White and black dots show the negative and positive chiral charges (*C*_*w*_), respectively. (**c**) Band structures of WTe_2_ along momentum space cut as defined in **a**. The four bands appeared in this panel are labelled as b1–b4, respectively. (**d**) Band structures of Mo_0.2_W_0.8_Te_2_ along momentum space cut as defined in **b**. We find an inversion between the b2 and b3 bands, giving rise to the Weyl nodes W1 and W2 with opposite chiral charges *C*_*w*_. (**e**) The *k* space separation between the pair of Weyl nodes W1 and W2 as a function of Mo doping *x*. (**f**) The energy difference between the extrema of the b2 and b3 bands as a function of Mo doping *x*. This characterizes the magnitude of the band inversion. (**g**) The energy offset between the pair of Weyl nodes W1 and W2 as a function of Mo doping *x*. The blue dashed lines are guides to the eye. (**h**) A schematic for the distribution of the Weyl nodes within a bulk BZ. All nodes are located on the *k*_*z*_=0 plane.

We now calculate the band structure of pure WTe_2_ throughout the bulk BZ based on the lattice constants reported in the paper by Brown^36^. Our results show that pure WTe_2_ has a continuous energy gap throughout the bulk BZ without any Weyl nodes. The *k* point that corresponds to the minimal gap is found to be close to the Γ−*Y* (Fig. 2a) axis. The minimal gap of WTe_2_ is 0.9 meV (Fig. 2c). We note that the discrepancy between our results and the results of Soluyanov *et al.*^35^ is due to the slightly different values of the lattice constants^36^,^39^. The lattice constants used by Soluyanov *et al.*^35^ were at low temperatures^39^. Thus the results^35^ better refelct the groundstate (*T*=0) of WTe_2_. We used the lattice constants at room temperatures^36^, so our results correspond to the state of WTe_2_ at elevated temperatures. The difference between our results and the results of Soluyanov *et al.*^35^ shows from another angle that WTe_2_ is very close to the phase boundary between the Weyl state and the fully gapped state. For many purposes, it is favourable to have the Weyl state in a material that is robust at elevated (room) temperatures. Here, we use the room temperature lattice constants for all of our calculations. We also note that the very small difference of the lattice constant value does not play a role except for undoped or very lightly doped samples, that is, *x*≤2%, where the separation of the Weyl nodes is beyond experimental resolution anyway.

We propose Mo-doped WTe_2_, Mo_*x*_W_1−*x*_Te_2_, as an experimental feasible platform to realize Weyl state in this compound. We have shown that pure WTe_2_ is very close to the phase-transition boundary. Therefore, the *k* splitting between the Weyl nodes would be beyond experimental resolution. On the other hand, another very recent paper proposed a Weyl state in pure MoTe_2_ (ref. 40), but, as shown above, the existence of the orthorhombic MoTe_2_ needs to be confirmed. By contrast, we show that the moderately Mo-doped WTe_2_ sample has a number of advantages, making it experimentally feasible. First, pure MoTe_2_ has many irrelevant bands crossing the Fermi level along the Γ−*X* and Γ−*Z* directions, whereas the band structure of moderately Mo-doped system is as clean as pure WTe_2_ (Fig. 1d–f). Second, as we will show below, a moderate Mo doping leads to a *k* space separation of the Weyl nodes that is similarly as large as that of pure MoTe_2_. Therefore, we propose the Mo-doped WTe_2_ as a better platform for studying Weyl physics.

### Doping dependency of the Weyl nodes

Figure 2e shows the evolution of the *k* space distance between a pair of Weyl nodes as a function of Mo concentration. Our calculation shows that a 2% Mo doping is sufficient to stabilize the system in the Weyl metal state. Also, the distance between the Weyl nodes increases rapidly at the small doping regime. At a moderate doping *x*=20%, the *k* space distance is found to be as large as 0.03 2*π*/*a*. As one further increases the doping concentration, the distance seems saturated. The distance is about 0.04 2*π*/*a* at *x*=40%. The energy difference between the pair of Weyl nodes is shown in Fig. 2g. In Fig. 2d we show the dispersion along the momentum space cut that goes through the direct pair of Weyl nodes as defined in Fig. 2b. It can be seen clearly that two singly generated bands, b2 and b3, cross each other and form the two Weyl nodes with opposite chiralities. We name the Weyl node at lower energy as W1 and the Weyl node at higher energy as W2. Another useful quantity is the energy difference between the extrema of these two bands. This characterizes the magnitude of the band inversion, as shown in Fig. 2f. It is interesting to note that, in contrast to the *k* space distance between the Weyl nodes (Fig. 2e), the energy difference between the Weyl nodes (Fig. 2g) and the band inversion energy (Fig. 2f) does not show signs of saturation as one increases the Mo concentration *x* up to 40%. In Fig. 2h, we show a schematic for the distribution of the Weyl nodes in Mo-doped WTe_2_. We observe a pair of Weyl nodes in each quadrant of the *k*_*z*_=0 plane. Thus in total there are four pairs of Weyl nodes on the *k*_*z*_=0 plane.

### Tunable Fermi arc surface states

**Figure 3:**
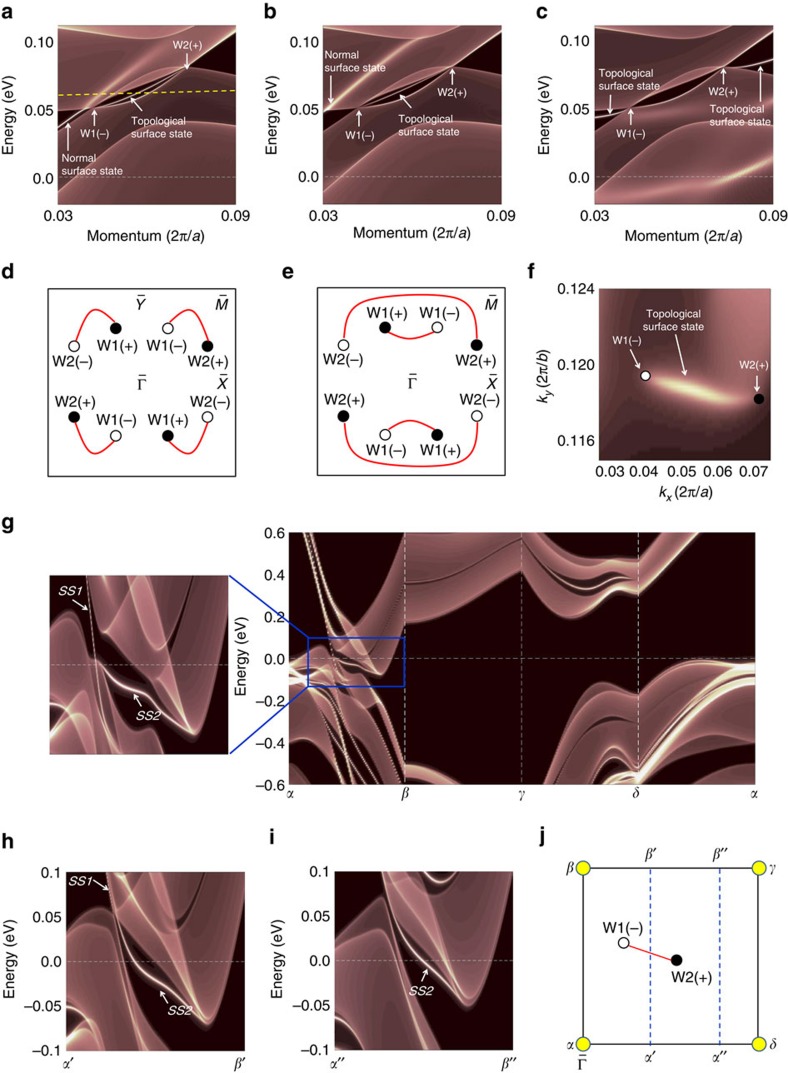
Tunable Fermi arc length and interconnectivity in Mo_*x*_W_1−*x*_Te_2_. (**a**) Surface and bulk band structure of Mo_0.2_W_0.8_Te_2_ along the momentum space cut that goes through a direct panel of Weyl nodes, W1(−) and W2(+). A topological Fermi arc surface state connects the Weyl nodes W1 and W2. A normal surface state avoids the Weyl nodes and merges into the bulk band continuum. (**b**) Same as in **a** but with the surface on-site energy increased by 0.02 eV. (**c**) Same as in **a** but with the surface on-site energy decreased by 0.11 eV. (**d**) Schematic of Fermi arc connectivity pattern for **a**,**b**. (**e**) Schematic of Fermi arc connectivity pattern for **c**. (**f**) Surface band structure on a varying-energy (*k*_*x*_, *k*_*y*_) map. Specifically, we choose a different energy on each (*k*_*x*_, *k*_*y*_) point, so that the there are no bulk states at all *k*_*x*_, *k*_*y*_ except at the locations of the eight Weyl nodes. This is possible because we know that the conduction and valence bands only cross at the eight Weyl nodes. (**g**) Surface and bulk band structure along the *k*—space trajectory *α*−*β*−*γ*−*δ* defined in **j**. (**h**,**i**) Surface and bulk band structure along the *k*—space trajectories *α*′−*β*′ and *α*′−*β*′ defined in **j**. (**j**) Schematic of a quadrant of the surface BZ, showing the *k*—space trajectories used in **g**–**i**.

A critical signature of a Weyl semimetal is the existence of Fermi arc surface states. We present calculations of the (001) surface states in Fig. 3. We choose the 20% Mo-doped system, Mo_0.2_W_0.8_Te_2_. Figure 3a shows the surface energy dispersion along the momentum space cut that goes through the direct pair of Weyl nodes, W1(−) and W2(+), which arises from a single band inversion. Here (+) and (−) denote positive and negative chiral charge, respectively. Our calculation (Fig. 3a) clearly shows the topological Fermi arc surface state, which connects the direct pair of Weyl nodes. The Fermi arc was found to terminate directly onto the projected Weyl nodes. In addition, we also observe a normal surface state, which avoids the Weyl node and merges into the bulk band continuum. Because the W1 and W2 Weyl nodes have different energies, and because W1 is a type II Weyl cone^35^, constant energy maps always have finite Fermi surfaces. Type II means that at the Weyl node energy, its constant energy contour consists of an electron and a hole pocket touching at a point, the Weyl node. Hence, visualizing Fermi arc connectivity in constant energy maps is not straightforward. Instead of a constant energy map, it is possible to use a varying-energy *k*_*x*_, *k*_*y*_ map, that is, Energy=*E*(*k*_*x*_, *k*_*y*_), so that there are no bulk states on this varying-energy map at all *k*_*x*_, *k*_*y*_ points except the Weyl nodes. Figure 3f shows the calculated surface and bulk electronic structure on such a varying-energy *k*_*x*_, *k*_*y*_ map in the vicinity of a pair of Weyl nodes. A Fermi arc that connects the pair of Weyl nodes can be clearly seen. We study the effect of surface perturbations. The existence of Weyl nodes and Fermi arcs are guaranteed by the system's topology, whereas the details of the surface states can change under surface perturbations. In order to do so, we change the surface on-site potentials of the system. Physically, the surface potentials can be changed by surface deposition or applying an electric field on the surface. Figure 3b shows the surface band structure with the surface on-site energy increased by 0.02 eV. We find that the normal surface state moves further away from the Weyl nodes, whereas the topological Fermi arc does not change significantly. Figure 3c shows the surface band structure with the surface on-site energy decreased by 0.11 eV. The normal surface states disappear. The Fermi arc also changes significantly. Instead of directly connecting the two Weyl nodes in Fig. 3a,b, a surface state stems from each Weyl node and disperses outside the window. We note that the surface states in Fig. 3c are still topological and are still arcs because they terminate directly onto the projected Weyl nodes. We illustrate the two types of Fermi arc connectivity in Fig. 3d,e. Figure 3d corresponds to the case in Fig. 3a,b, where a Fermi arc directly connects the pair of Weyl nodes in a quadrant. Figure 3e corresponds to Fig. 2d. In this case, Fermi arcs connect Weyl nodes in two different quadrants across the 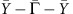 line. The normal surface states do not exist necessarily as they can be removed by tuning the on-site energy as shown in Fig. 3a,b. The nontrivial topology in a Weyl semimetal requires that there must be Fermi arc(s) terminating onto each projected Weyl node with a non-zero projected chiral charge and that the number of Fermi arcs associated with a projected Weyl node must equal its projected chiral charge. On the other hand, the pattern of connectivity can vary depending on details of the surface. The observed different Fermi arc connectivity patterns as a function of surface on-site potential provide an explicit example of both the constraints imposed and the freedoms allowed to the Fermi arc electronic structure by the nontrivial topology in a Weyl semimetal.

We further study the surface states via bulk boundary correspondence. We note that except Fig. 3b–e, all other figures correspond to the case without additional changes to the on-site energy. Specifically, we choose a closed loop in (*k*_*x*_, *k*_*y*_) space as shown in Fig. 3j. As we mentioned above, the conduction and valence bands only touch at the eight Weyl nodes. Thus as long as the loop chosen does not go through these Weyl nodes, there is a continuous bulk energy gap along the loop. In the bulk BZ, the chosen rectangular loop corresponds to a rectangular pipe along the *k*_*z*_ direction. Then topological band theory requires that the net chiral charge of the Weyl nodes that are enclosed by the pipe equals the Chern number on this manifold, which further equals the net number of chiral edge modes along the loop. For example, the rectangular loop *α*−*β*−*γ*−*δ* in Fig. 3j encloses a W1(−) and a W2(+), which leads to a net chiral charge zero. The energy dispersion along this rectangular loop is shown in Fig. 3g. It can be seen that the bands are fully gapped without any surface states along *β*−*γ*−*δ*−*α*. Along *α*−*β*, there are two surface states (SS1 and SS2), both of which connect the band gap. Interestingly, we note that these two surface bands are counter-propagating although they seem to have the same sign of Fermi velocity. This is because the continuous energy gap *α*−*β* is highly ‘tilted'. If we ‘tilt' the energy gap back to being horizontal, then it can be clearly seen that the two surface bands are counter-propagating, which means that the net number of chiral edge modes is zero. Similarly, we can choose other loops. For example, we choose another rectangular loop *α*′−*β*′−*γ*−*δ* that encloses only the W2(+) Weyl node. Because there are no surface states along the two horizontal edges and the vertical edge to the right, we only need to study the vertical edge to the left, that is the *α*′−*β*′. The enclosed net chiral charge is +1, which should equal the net number of chiral edge mode along *α*′−*β*′. The band structure along this line is shown in Fig. 3h. We see that while the surface band SS1 still connects across the band gap, SS2 starts from and ends at the conduction bands. Therefore, SS1 contributes one net chiral edge mode, whereas SS2 contributes zero net chiral edge mode. Hence there is one net chiral edge mode along the this rectangular loop. By the same token, we can choose the loop *α*″−*β*″−*γ*−*δ*, which does not enclose any Weyl node. Consistently, as shown in Fig. 3i, along *α*″−*β*″, SS1 does not appear along this line and SS2 does not connect across the band gap. Hence the net number of chiral edge mode is also zero along this rectangular loop.

**Figure 4:**
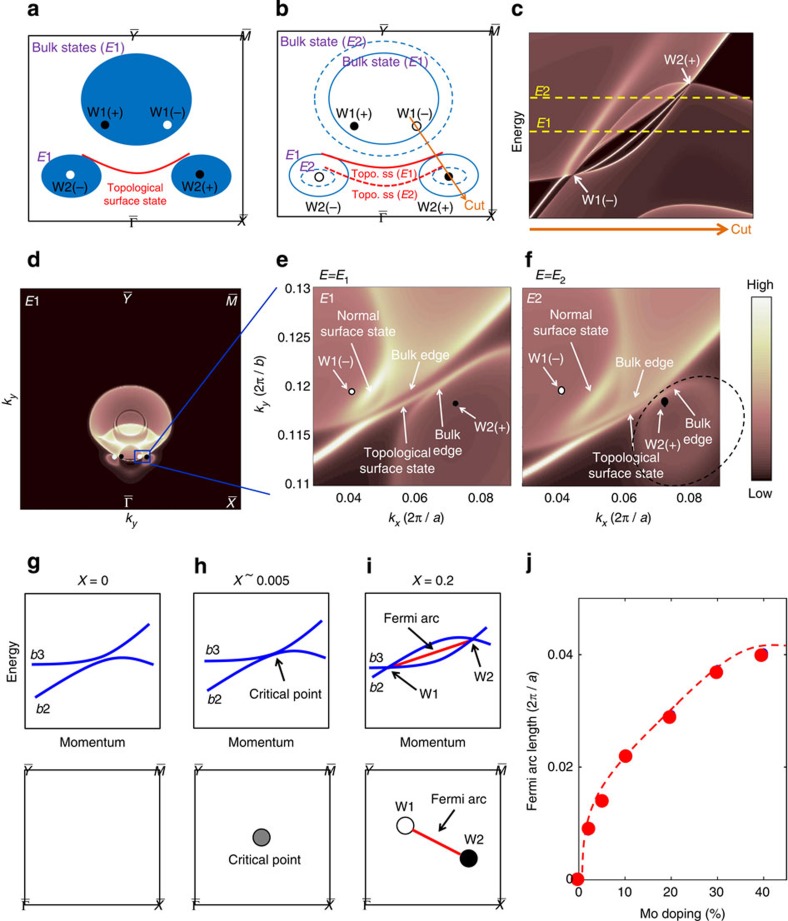
Iso-energy contour interconnectivity on the surface of Mo_*x*_W_1−*x*_Te_2_. (**a**) Schematic illustration of the surface and bulk electronic structure on a constant energy *E*_1_. The shaded areas represent the projected bulk bands, whereas the red line show the surface states. (**b**) Schematic illustration of the surface and bulk electronic structure on two energies *E*_1_ and *E*_2_. The band structure on *E*_1_ (*E*_2_) are shown by the solid (dotted) lines. The energies *E*_1_ and *E*_2_ are defined in **c**. (**c**) Band structure along a *k* space cut that goes through the direct pair of Weyl nodes, W1(−) and W2(+). The dotted lines denote the energies *E*_1_ and *E*_2_ with respect to the W1 and W2 Weyl nodes. (**d**) Calculated surface and bulk electronic structure on at the energy *E*_1_ over the top half of the surface BZ. (**e**) A zoomed-in view of **d** for the area highlighted by the blue box. (**f**) The same as **e** but at the energy *E*_2_. (**g**–**i**) Schematic band diagram to show the evolution of the Mo_*x*_W_1−*x*_Te_2_ system as a function of Mo concentration *x*. (**j**) The length of the Fermi arc as a function of Mo concentration *x*. The arc length equals the *k* space separation of the Weyl nodes.

We study the constant energy contours of the surface states. We emphasize that (i) there is a significant energy offset between the W1 and W2 Weyl nodes, and that (ii) the W1 Weyl cones are the type II Weyl cone^35^. These two properties are very different from the ideal picture, where all Weyl cones are normal rather than type II and their nodes are all at the same energy. We show below that these two properties make the surface states' constant energy contours quite different from what one would expect naively. Figure 4d shows the calculated constant energy contour within the top half of the surface BZ at energy *E*_1_, which is between the W1 and W2 nodes in energy. More constant energy contours at other energies are shown in the Supplementary Fig. 1 and Supplementary Note 1. We see three bulk pockets. The corresponding schematic is shown in Fig. 4a. Specifically, we see a big pocket closer to the 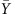 point, which encloses two W1 Weyl nodes with opposite chiral charges. We also see two separate small pockets closer to the 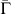, each of which encloses a W2 Weyl node. As for the surface states, from Fig. 4d,e we see a surface state band that connects the two small pockets, each of which encloses a W2 Weyl node. This is quite counter-intuitive because we know that the Fermi arc connects the direct pair of Weyl nodes, namely a W1(−) and a W2(+) or vice versa. We show that there is no discrepancy. Specifically, we show that the surface band seen in the constant energy contours is exactly the Fermi arc that connects the W1(−) and the W2(+) Weyl nodes seen in Fig. 4c. To do so, we consider the constant energy contours at two different energies, *E*_1_ and *E*_2_. According to the energy dispersion (Fig. 4c), we see that the big bulk pocket in the constant energy map is electron-like, while the two small bulk pockets are hole-like. Thus as we increase the energy from *E*_1_ to *E*_2_, the big pocket should expand, whereas the two small pockets should shrink, as shown in Fig. 4b. The surface state band keeps connecting the two small pockets as one changes the energy. This evolution is shown by real calculations in Fig. 4e,f. The orange line in Fig. 4b connects the W1(−) and W2(+) Weyl nodes. At each energy, the surface state band crosses the orange line at a specific *k* point. By picking up the crossing points at different energies, we can reconstruct the Fermi arc that connects the W1(−) and W2(+) Weyl nodes shown in Fig. 4c. Therefore, from our systematic studies above, we show that the Fermi arc connectivity means the pattern in which the surface state connects the Weyl nodes. This is defined on a varying-energy (*k*_*x*_, *k*_*y*_) map where the chosen *E*(*k*_*x*_, *k*_*y*_) map crosses the bulk bands only at the Weyl nodes. If there is no significant energy offset between Weyl nodes and if all Weyl cones are normal rather than type II, then the connectivity can also be seen in a constant energy contour. However, in our case here, one needs to be careful with the simplified ideal picture, that is, to study the Fermi arc connectivity from the constant energy contour. Because of the energy offset between the Weyl nodes and because of the existence of type II Weyl cones, how surface bands connect different bulk pockets in a constant energy contour does not straightforwardly show the Fermi arc connectivity.

## Discussions

We discuss the tunability of the length of the Fermi arcs as a function of Mo concentration *x* in our Mo_*x*_W_1−*x*_Te_2_ system. The undoped *x*=0 sample is fully gapped according to our calculations (Fig. 4g). A very small Mo concentration (∼0.5%) will drive the system to the critical point, where the conduction and valence bands just touch each other Fig. 4h. The length of the Fermi arc is zero, and hence the system is at the critical point. As one further increases the Mo concentration *x*, the touching point splits into a pair of Weyl nodes with opposite chiralities (Fig. 4i). The Weyl nodes are connected by a Fermi arc. A way to gap the system without breaking any symmetry is to annihilate pairs of Weyl nodes with opposite chiralities. In order to do so, one needs to overcome the momentum space separation between the Weyl nodes to bring them together in the *k* space. Thus the length of the Fermi arc that directly connects the Weyl nodes by the shortest distance provides a measure of the system's topological strength. Such a tunability is not known in the TaAs class of Weyl system. We also compare our proposal to previous candidates^9^,^41^,^42^,^43^,^44^,^45^,^46^,^47^, where analogous tunability was proposed. We emphasize that the key difference is the experimental feasibility of the proposals. For example, refs 41, 42, 43 used hypothetic crystal structures which have never been synthesized. Reference 9 requires a ferromagnetic normal insulator that is lattice matched with the known topological insulators, which are currently unknown. Reference 44 requires external magnetic fields and hence ARPES cannot be done. References 46,47 require external pressure and cannot be realized at ambient conditions. As stressed in the TaAs prediction^19^, in order to have a robust material candidate, following aspects are crucial: first, the proposal can be realized at ambient conditions with a realistic crystal structure that has been successfully synthesized. Second, there is no need to align the magnetic domains as in the ferromagnetic compounds; Third, the proposed candidate does not need fine-tuning of the chemical composition (in our case, any composition within the the wide range of 3 to 40% works). Fourth, the separation of Weyl nodes is large enough for experimental observation, which is not the case for WTe_2_. These aspects were crucial for the experimental discovery of TaAs^19^,^21^ and are also satisfied by our prediction of Mo_*x*_W_1−*x*_Te_2_ here. We hope that our prediction can provide another material realization which is critically needed for this rapidly developing field.

## Methods

### Computational details

We computed the electronic structures using the projector augmented wave method^48^,^49^ as implemented in the VASP package^50^ within the generalized gradient approximation schemes^51^. For WTe_2_, experimental lattice constants were used^36^. For MoTe_2_, we assumed that it has the same crystal structure as WTe_2_ and calculated the lattice constants self-consistently (*a*=6.328 Å, *b*=3.453 Å, *c*=13.506 Å). A 8 × 16 × 4 MonkhorstPack *k*-point mesh was used in the computations. The spin-orbit coupling effects were included in calculations. In order to systematically calculate the surface and bulk electronic structure, we constructed a tight-binding Hamiltonian for both WTe_2_ and MoTe_2_, where the tight-binding model matrix elements were calculated by projecting onto the Wannier orbitals^52^,^53^,^54^, which used the VASP2WANNIER90 interface^55^. We used W (Mo) *s* and *d* orbitals and Te *p* orbitals to construct Wannier functions without using the maximizing localization procedure. The electronic structure of the samples with finite dopings was calculated by a linear interpolation of tight-binding model matrix elements of WTe_2_ and MoTe_2_. Since tight-binding parameters contained all important information such as lattice constants and atomic bonding strength, an interpolation presumably covered all systematic changes of the electronic structure between the two end points. Therefore, this approach is highly reliable and effective for studying iso-valence substitution/doping. Previously, we used the same method to investigate a similar iso-valence substitution in the BiTlSe_1−*x*_S_*x*_ and predicted a critical point of *x*_*c*_=0.48 that was in excellent agreement with experimental finding of *x*_*c*_=0.5 (ref. 56). Also, since we mainly studied small Mo doping, for example, Mo=20% that is close enough to the end compound, a linear interpolation should be a good approximation. The surface state electronic structure was calculated by the surface Green's function technique, which computes the spectral weight near the surface of a semi-infinite system.

## Additional information

**How to cite this article:** Chang, T.-R. *et al.* Prediction of an arc-tunable Weyl Fermion metallic state in Mo_*x*_W_1−*x*_Te_2_. *Nat. Commun.* 7:10639 doi: 10.1038/ncomms10639 (2016).

## Supplementary Material

Supplementary Information Supplementary Figure 1 and Supplementary Note 1
