# Supplementary material for: Prediction of an arc-tunable Weyl Fermion metallic state in MoxW1−xTe2
Source: Nat Commun. 2016 Feb 15;7:10639. doi: 10.1038/ncomms10639 (PMC4756349; doi:10.1038/ncomms10639)
Supplement: Supplementary Information — Supplementary Figure 1 and Supplementary Note 1 [file ncomms10639-s1.pdf]

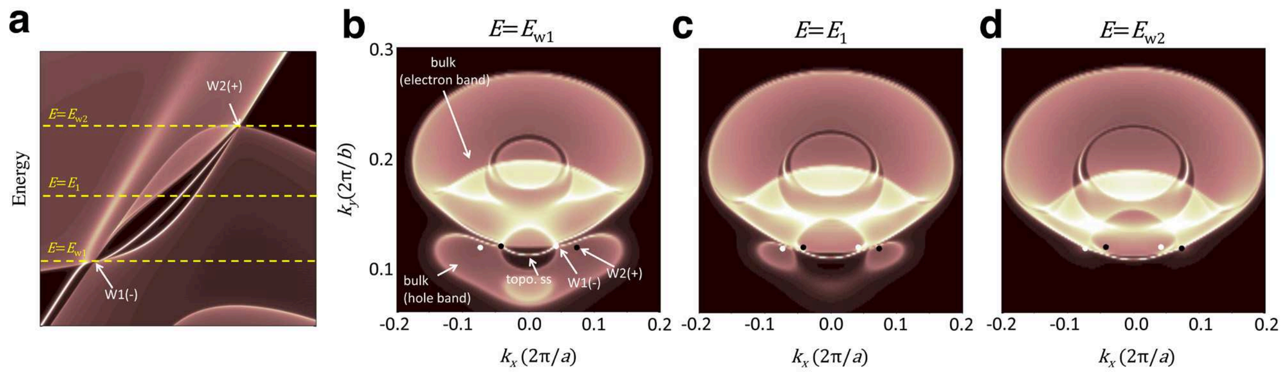

Supplementary Figure 1: **Constant energy contours at different binding energies. a,** Band structure along a k space cut that goes through the direct pair of Weyl nodes, W1(-) and W2(+). **b,** Constant energy contours at the energy E_{w1}. **c,** Constant energy contours at the energy E_1. **d,** Constant energy contours at the energy E_{w2}.

Supplementary Note 1: Constant energy contours at different binding energies

Here we show constant energy contours of $Mo_{0.2}W_{0.8}Te_2$ at different binding energies (Supplementary Fig. 1). We only show very high resolution calculations at k points around the Γ point because there are no states near the surface Brillouin zone boundaries. The constant energy contour at the energy E_1 is the same as Fig. 4**d** in the main text.
